# Identification and characterization of stem cell secretome-based recombinant proteins for wound healing applications

**DOI:** 10.3389/fbioe.2022.954682

**Published:** 2022-07-22

**Authors:** Ji Hyun Kim, Denethia S. Green, Young Min Ju, Mollie Harrison, J. William Vaughan, Anthony Atala, Sang Jin Lee, John D. Jackson, Cory Nykiforuk, James J. Yoo

**Affiliations:** ^1^ Wake Forest Institute for Regenerative Medicine, Wake Forest School of Medicine, Winston-Salem, NC, United States; ^2^ Emergent BioSolutions, Winnipeg, MB, Canada

**Keywords:** stem cell secretome, conditioned medium, recombinant proteins, wound healing, skin regeneration, tissue engineering and regenerative medicine

## Abstract

Stem cells have been introduced as a promising therapy for acute and chronic wounds, including burn injuries. The effects of stem cell-based wound therapies are believed to result from the secreted bioactive molecules produced by stem cells. Therefore, treatments using stem cell-derived conditioned medium (CM) (referred to as secretome) have been proposed as an alternative option for wound care. However, safety and regulatory concerns exist due to the uncharacterized biochemical content and variability across different batches of CM samples. This study presents an alternative treatment strategy to mitigate these concerns by using fully characterized recombinant proteins identified by the CM analysis to promote pro-regenerative healing. This study analyzed the secretome profile generated from human placental stem cell (hPSC) cultures and identified nine predominantly expressed proteins (ANG-1, FGF-7, Follistatin, HGF, IL-6, Insulin, TGFβ-1, uPAR, and VEGF) that are known to contribute to wound healing and angiogenesis. These proteins, referred to as s (CMFs), were used in combination to test the effects on human dermal fibroblasts (HDFs). Our results showed that CMF treatment increased the HDF growth and accelerated cell migration and wound closure, similar to stem cell and CM treatments. In addition, the CMF treatment promoted angiogenesis by enhancing new vessel formation. These findings suggest that the defined CMF identified by the CM proteomic analysis could be an effective therapeutic solution for wound healing applications. Our strategy eliminates the regulatory concerns present with stem cell-derived secretomes and could be developed as an off-the-shelf product for immediate wound care and accelerating healing.

## Introduction

Burns and full-thickness wounds present a major clinical challenge for patients, even after treatment. In the US, nearly 15% of Medicare beneficiaries (roughly 8.2 million) are affected by chronic non-healing wounds, at an annual healthcare cost between $28.1–$31.7 billion; this estimate includes only the cost to Medicare and excludes the costs charged to private insurers (Nussbaum et al., 2018; Sen, 2019). Over the years, numerous technological advances have been made in treating acute and chronic non-healing wounds; however, healing rates remain below 50% (Cherry et al., 2008; Sen et al., 2009; Sun et al., 2014). While split-thickness autologous skin grafts remain the standard procedure for burns and chronic wounds, this treatment method is limited by the donor tissue availability and donor site morbidity (Robson et al., 1992; Yang et al., 1992; Kurd et al., 2009; Sun et al., 2014). Alternative treatments such as allografts and biomaterial-based skin substitutes have been used, but frequent graft failure has remained a problem (Kurd et al., 2009; Sen et al., 2009). While these advances have shown some levels of improvement, none has achieved the long-term success that autologous skin grafts offer. There is a dire need to develop rapid, easy-to-use, and durable treatment options to accelerate the healing process, protect from wound infections, and promote complete non-fibrotic wound closure.

Stem cell-based therapies offer immense promise for wound treatments because they can be readily expanded under GMP manufacture to provide coverage for large wounds and are known to accelerate the wound healing (Kanji and Das, 2017; Martinello et al., 2018; Nourian Dehkordi et al., 2019; Huang et al., 2020). Of the stem cell sources, human placental stem cells (hPSCs) have gained increasing attention recently due to their low immunogenicity and capacity to immunomodulate wound beds, thereby signaling re-epithelialization with limited fibrosis (Abumaree et al., 2013; Kong et al., 2013; Abd-Allah et al., 2015; Wang et al., 2016). Many studies have shown that hPSCs are useful for promoting healing in acute full-thickness wound care (Kong et al., 2013; Abd-Allah et al., 2015; Wang et al., 2016). It is believed that their therapeutic effects are achieved through the secretory proteins produced by these cells that stimulate growth by enlisting and directing native regenerative resources toward the repair (Kong et al., 2013; Abd-Allah et al., 2015). These secretory proteins present in a conditioned medium (CM) are thought to promote angiogenesis and re-epithelialization (Kong et al., 2013; Abd-Allah et al., 2015; Du et al., 2016).

Based on literature reports, stem cell CM (secretome) has emerged as an alternative option to cell-based therapy. The CM produced by stem cell cultures contains numerous bioactive molecules such as cytokines, growth factors, and extracellular matrix (ECM) proteins that enhance cell viability, proliferation, migration, and immunomodulation (Pawitan, 2014). The treatment effects of CM on tissue regeneration and repair have been demonstrated in multiple tissues and organ systems, including kidney (Yim et al., 2019), heart (Timmers et al., 2007), spinal cord (Cantinieaux et al., 2013), corpora (Matz et al., 2020), and bone (Osugi et al., 2012). Recently, several preclinical studies have reported wound healing effects for CM derived from bone marrow mesenchymal stem cells (MSCs) (Chen et al., 2014), umbilical cord MSCs (Li et al., 2017), amniotic fluid stem cells (AFSCs) (Jun et al., 2014), and hPSCs (Du et al., 2016). Although the CM treatment has demonstrated enormous potential for tissue regeneration and wound healing, many issues remain, requiring extensive cell expansion, variation in CM content, and unknown and inhibitory trophic factors present in CM (Tran and Damaser, 2015). More importantly, safety concerns have risen as a major limitation to using this approach due to its uncharacterized biochemical content.

In our previous studies, we have demonstrated the therapeutic effects of human fetal stem cell-derived CM obtained from amniotic fluid or placenta (hAFSC and hPSC) and identified critical proteins that contribute to tissue regeneration using the proteomics analysis of the CM (Skardal et al., 2012; Yim et al., 2019; Matz et al., 2020). In one study, we confirmed the effects of hAFSC increasing wound closure rates and angiogenesis. The hAFSC-derived CM, containing key growth factors such as vascular endothelial growth factor (VEGF), basic fibroblast growth factor (bFGF), and hepatocyte growth factor (HGF), support the treatment effects of hAFSC (Skardal et al., 2012). In other studies, the delivery of hPSC-derived CM improved the morphological and functional recovery of damaged tissues by enhancing cell proliferation, reducing apoptotic cell death, and promoting angiogenesis, showing similar therapeutic effects to the hPSC transplantation (Yim et al., 2019; Matz et al., 2020). We confirmed that the proteins contained in hPSC-CM are critical for tissue protection and regeneration [e.g., angiopoietin (ANG), HGF, follistatin] (Yim et al., 2019). In addition, these protein concentrations were higher than in CM derived from other cell types, including hAFSC, adipose-derived stem cells, and endothelial cells (Matz et al., 2020). These results indicate that key proteins secreted by hPSC may play a crucial role in promoting tissue regeneration. The results from our previous studies support the possibility of using defined proteins contained in stem cell-derived CM for wound healing treatment as an alternative to stem cells or CM.

This study developed a strategy to identify stem cell secretome-based recombinant proteins (referred to as conditioned medium factor (CMF)) for wound healing applications. The specific protein combination determined by the stem cell-derived secretome analysis could be developed as a therapy, as a form of recombinant protein cocktail. This strategy of using identified recombinant proteins has many advantages over using CM. Using a defined composition and dosage of the recombinant protein cocktail (CMF) eliminates the need for extensive cell culture and CM collection. It would maintain a therapeutically consistent product, thereby providing an off-the-shelf option that could be useful for the acute phase of the wound and burn care.

In the present study, we analyzed the secretome profile of hPSC-derived CM cultivated in normoxia and hypoxia by proteomics and identified the proteins that are effective in wound healing and regeneration. Several studies reported that stem cells secrete anti-apoptotic, pro-proliferative, and pro-angiogenic factors under hypoxic conditions (Das et al., 2010; Chen et al., 2014; Jun et al., 2014). The hPSC secretome profile in hypoxia can be different from that in normoxia. Given that wound beds are typically hypoxic, it was necessary to investigate the secretome profile of hPSC-derived CM in the hypoxic environment. We developed a stem cell secretome-based recombinant protein cocktail (CMF) that synergistically affects the regenerative capacities of the human dermal fibroblasts (HDF) and endothelial cells responsible for wound healing. This study focused on investigating the CMF treatment effects using HDF. We hypothesized that the CMF treatment would enhance HDF growth and migration and prevent apoptotic cell death, which are the characteristics of cellular response in wound healing. We optimized the concentration of CMF in terms of HDF growth. To determine the effectiveness of the protein combination, HDF growth under protein cocktail treatment was compared with those of each protein treatment. The treatment efficacy of CMF on HDF proliferation, anti-apoptosis, and migration was evaluated and compared with hPSC-derived CM treatment. In addition, the CMF treatment effects on promoting angiogenesis were assessed using an *in ovo* chick chorioallantoic membrane (CAM) assay. The effect of CMF on endothelial cell growth was evaluated.

## Materials and methods

### Cell culture

hPSCs isolated from donated human placenta were obtained from the Manufacturing Development Center at Wake Forest Institute for Regenerative Medicine (IRB# 00056941) (Zhang et al., 2016; Yim et al., 2019). hPSCs (passage 6–7) were cultured in hPSC growth medium (hPSC-GM) containing 65% of Alpha Minimum Essential Medium (ά-MEM; Gibco, Amarillo, TX), 17% Amniomax C100 Basal Medium (Gibco), 15% Fetal Bovine Serum (FBS; Gibco), 2% Aminomax Supplement (Gibco), 1% Glutamax (Gibco), and 2.5 μg/ml Gentamicin (Gibco). HDFs isolated from donated infant foreskin were obtained from the Wake Forest Institute for Regenerative Medicine Skin Team (IRB# 00007586) (Jorgensen et al., 2020). HDFs were cultured in Dulbecco’s Modified Eagle Medium (DMEM; Cytiva; Logan, UT, United States) containing 10% FBS and 1% Penicillin/Streptomycin (P/S; Fischer Scientific, Waltham, MA) (HDF-GM) (passage 5–7) (Jorgensen et al., 2020). The medium was changed every 2–3 days. Human umbilical vein endothelial cell (HUVECs) (Lonza, Basel, Switzerland) were cultured in HUVEC growth medium (HUVEC-GM) containing Endothelial Cell Basal Medium-2 (EBM-2; Lonza) and EBM-2 SingleQuots (EBM-2-SQ; 2% Fetal Bovine Serum, 0.04% Hydrocortisone, 0.4% hFGF-B, 0.1% VEGF, 0.1% R3-IGF-1, 0.1% Ascorbic Acid, 0.1% hEGF, 0.1% GA-1000, 0.1% Heparin; Lonza) (passage 3). The medium was changed every 2–3 days.

### Preparation of hPSC-derived conditioned medium and proteomics analysis

**FIGURE 1:**
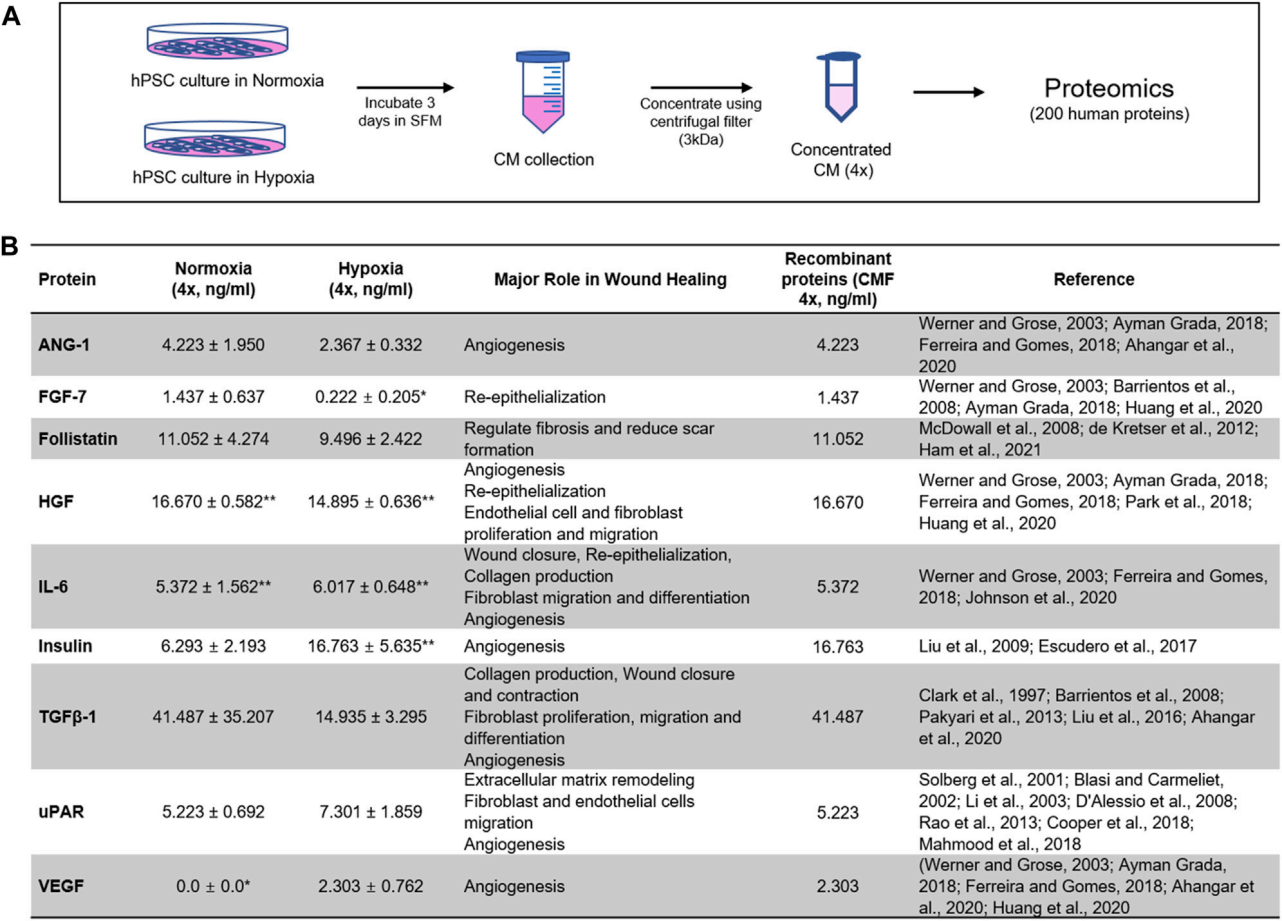
Proteomics analysis of stem cell-derived secretome. Identification of highly expressed human proteins by proteomics of human placental stem cell (hPSC)-derived conditioned medium (CM) in normoxia (21% O_2_) and hypoxia (1% O_2_). **(A)** CM collected from 3-day hPSC culture in serum-free medium (SFM) is concentrated 4× with 3 kDa centrifugal unit, and 200 human proteins are evaluated with proteomics analysis (*n* = 3). **(B)** Nine proteins highly expressed in normoxia and hypoxia with major roles in wound healing are identified. The composition of recombinant proteins [condition medium factor (CMF 4×)] for wound healing applications. *LOD (value below the limit of detection), **MAX (value above the highest standards). ANG-1: Angiopoietin-1; FGF-7: Fibroblast growth factor-7; HGF: Hepatocyte growth factor; IL-6: Interleukin-6; TGFβ-1: Transforming growth factor beta-1; uPAR: Urokinase receptor; VEGF: Vascular endothelial growth factor.

hPSCs (10,000 cells/cm^2^) were plated in a 15 cm culture dish and cultured overnight in hPSC-GM. The cells were re-fed with serum-free hPSC medium (hPSC-SFM; hPSC-GM without FBS and Amniomax Supplement) and incubated in normoxia (21% O_2_) and hypoxia (1% O_2_) (Hypoxia C-chamber, Biospherix, Parish, NY, United States) conditions for 3 days, respectively. hPSC-CM obtained from normoxia (hPSC-CM-N) and hypoxia (hPSC-CM-H) were collected and centrifuged at 1500 rpm for 5 min. The supernatant was collected to eliminate cellular debris. The hPSC-CM was concentrated 4-fold (4×) using Amnicon Ultra-15 Centrifugal Filter Units with a molecular cutoff of 3 kDa (Millipore Sigma, Germany). The concentrated hPSC-CM was frozen and stored at −80°C until use. For proteomics, hPSC-CM was collected from 3 different donors. The 4-fold (4×) concentrated hPSC-CM was analyzed for 200 human protein content using Quantibody Human Cytokine Array Q4000 (Quantibody quantitative multiplex ELISA array, catalog # QAH-CAA-4000: Raybiotech, Norcross, GA) **(** Figure 1A **)**.

### Condition medium factor concentration test

**FIGURE 2:**
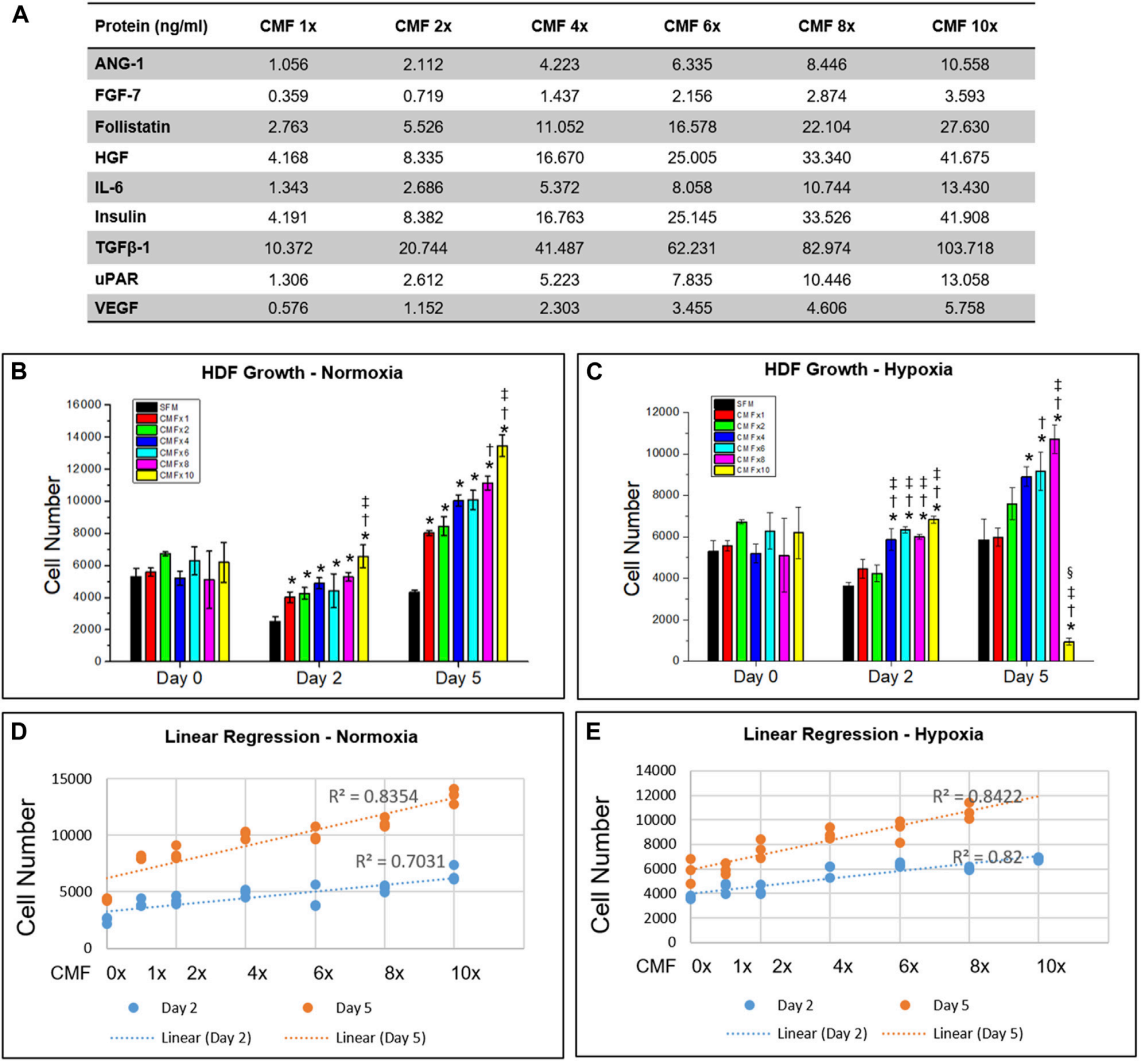
Optimization of CMF concentration. Human dermal fibroblasts (HDF) are treated with SFM (CMF 0×) and various CMF (nine protein combination: ANG-1, FGF-7, Follistatin, HGF, IL-6, Insulin, TGFβ-1, uPAR, and VEGF) concentrations (CMF 1×, 2×, 4×, 6×, 8×, and 10×) **(A)**, and cell growth is measured using Alamar Blue assay. **(B,C)** Cell number cultured in normoxia **(B)** and hypoxia **(C)**. **(D,E)** Linear regression relation between CMF concentration and cell number in normoxia **(D)** and hypoxia **(E)**. In hypoxia, CMF 10× data is excluded. The results indicate that CMF increases cell growth in a dose-dependent manner but not in concentrations greater than 6×. Overall, CMF at a concentration of 6× is the optimal concentration to enhance HDF growth (*n* = 3, duplicate measure, two-way ANOVA and Tukey’s test, (B) * *p* < 0.05 with SFM, ^†^ *p* < 0.05 with CMF 1×, ^‡^ *p* < 0.05 with CMF 2×, 4×, and 6×, NS: *p* > 0.05, **(C)** *p < 0.05 with SFM. ^†^ *p* < 0.05 with CMF 1×. ^‡^ *p* < 0.05 with CMF 2×, ^§^ *p* < 0.05 with CMF 4×, 6×, and 8×, NS: *p* > 0.05).

Nine proteins effective for wound healing were selected based on the normoxic hPSC-CM (hPSC-CM-N) and hypoxic hPSC-CM (hPSC-CM-H) analysis. The proteins that promote wound healing and angiogenesis and were expressed at higher than normal serum levels in hPSC-CM-N and hPSC-CM-H were selected. The proteins possessing inhibitory effects were excluded from the selection. The combination of the nine proteins was prepared using recombinant human proteins (CMF). The CMF includes 1) ANG-1 (R&D Systems, Minneapolis, MN), 2) FGF-7 (R&D Systems), 3) Follistatin (Sigma-Aldrich, St. Louis, MO), 4) HGF (Sigma Aldrich), 5) Interleukin-6 (IL-6; R&D Systems), 6) Insulin (Sigma Aldrich), 7) Transforming growth factor beta-1 (TGFβ-1; R&D Systems), 8) Urokinase receptor (uPAR; R&D Systems), and 9) VEGF (R&D Systems) **(** Figure 1B **)**. The concentration of ANG-1, FGF-7, Follistatin, HGF, IL-6, TGFβ-1, and uPAR was determined to be equivalent to the concentration in hPSC-CM-N, because the protein concentration in hPSC-CM-N and hPSC-CM-H was not statistically different (*p* > 0.05). The Insulin and VEGF concentration was determined to the equivalent to the concentration in hPSC-CM-H, because these protein concentrations were higher in hPSC-CM-H. To determine the optimal CMF concentration, various concentrations of CMF were tested using HDFs. HDFs (5000 cells/well) were plated in 48 well plates in HDF-GM overnight. HDFs were treated with HDF-serum free medium (HDF-SFM; DMEM and 1% P/S), CMF 1×, CMF 2×, CMF 4×, CMF 6×, CMF 8×, and CMF 10× and cultured under normoxia and hypoxia, respectively **(** Figure 2A **)**. The concentration of each protein in the CMF 4× was equivalent to that in the 4× concentrated CM obtained from the hPSC-CM analysis (Figure 1B). The concentrations of each protein in all other CMF groups were calculated from the analyzed CM protein concentration. After treatment for 0, 2, and 5 days, the Alamar Blue assay was performed according to the manufacturer’s instruction (Invitrogen; Waltham, MA). Briefly, the cells were incubated in Alamar Blue assay reagent (1:10 dilution) for 2 h at 37°C. The incubated reagent was transferred to clear flat bottom 96 well plates, and fluorescence was measured at a wavelength of 560 and 590 nm (excitation and emission) (Spectra Max M5 multiplate reader, Molecular Devices, San Jose, CA, United States) (*n* = 3 per group and time point, duplicate measure). The measured optical density (OD) value was converted to cell number using a standard curve. To develop the standard curve between OD value and known HDF cell number, HDFs were seeded at a density of 0, 1563, 3125, 6250, 12,500, 25,000, 50,000, and 100,000 cells/well and cultured overnight. The OD value corresponding to the cell number was obtained using the Alamar Blue assay (*n* = 3).

### Comparison of cell growth between single protein and combinatorial proteins

The effectiveness of the recombinant protein combination (CMF) compared with individual proteins on cell growth was evaluated using an Alamar Blue assay. HDFs (5000 cells/well) were cultured overnight in HDF-GM in 48 well plates. The cells were treated with HDF-GM, HDF-SFM, CMF composed of nine recombinant human protein cocktails (ANG-1, FGF-7, Follistatin, HGF, IL-6, Insulin, TGFβ-1, uPAR, VEGF), or individual proteins. In this study, CMF 6× was tested because the CMF 6× was determined as the optimal CMF concentration in the above CMF concentration study. The concentration of individual protein was equivalent to that in the CMF 6×. After the treatment, the cells were cultured in normoxia and hypoxia, respectively. On days 0, 2, and 5, the Alamar Blue assay was performed (*n* = 3 per group and time point, duplicate measure). The measured OD value was converted to cell numbers using a standard curve described above. The cell number of each group was normalized to the day 0 cell number.

### Effects of condition medium factor on human dermal fibroblast growth

The effect of CMF on HDF growth was compared with hPSC-CM and co-culture with hPSC. HDFs (10,000 cells/well) were plated in 24 well plates overnight in HDF-GM and then treated with HDF-GM, HDF-SFM, hPSC-CM-N 6× or hPSC-CM-H 6×, and CMF 6×. For the co-culture group, hPSC (0.315 × 10^6^ cells) were plated in the transwell insert (Transwell Permeable Supports; Corning, NY) in hPSC-GM. hPSC seeding density used for the co-culture and CM 6× collection was the same. Following overnight culture, the hPSC-seeded insert was combined with the HDF-seeded 24 well plates and cultured in HDF-SFM. For the hPSC-CM group, the CM was concentrated 10-fold (10×) using Amnicon Ultra-15 Centrifugal Filter Units and diluted to 6× concentration with HDF-SFM. Test samples were treated with CM collected under normoxia (hPSC-CM-N) and CM collected under hypoxia (hPSC-CM-H), in normoxia and hypoxia respectively. The CM samples were pooled from the donors that were tested in the proteomics study. All test samples were cultured in normoxia and hypoxia, and an Alamar Blue assay was performed on days 0, 2, and 5. The OD value of each sample was converted to the cell number using a standard curve. The cell number of each group and time point was normalized to the cell number at day 0. The standard curve was developed with the seeding density of 0, 3125, 6250, 12,500, 25,000, 50,000, 100,000, and 200,00 cells/well in 24 well plates (*n* = 3 per group and time point, duplicate measure).

### Proliferating nuclear cell antigen and cleaved caspase-3 ELISA assay

Apoptosis and proliferation of HDFs were evaluated with Cleaved Caspase-3 (CC3) ELISA Kit (Abcam, Cambridge, MA, United States) and Proliferating Nuclear Cell Antigen (PCNA) ELISA Kit (Abcam) (*n* = 3). HDFs (0.75 × 10^6^ cells/10 cm dish) were cultured overnight in HDF-GM and then treated with HDF-GM, HDF-SFM, hPSC-CM-N 6× or hPSC-CM-H 6×, and CMF 6× for 3 days in normoxia and hypoxia, respectively. HDF-SFM was used to dilute hPSC-CM 10× stock to hPSC-CM 6×. The treatment medium was decanted, and HDFs were lysed by incubating the HDFs in 1× RIPA buffer (Cell Signaling Technology, Danvers, MA) with a protease inhibitor cocktail (1:100; Cell Signaling Technology) for 15 min on ice. Lysed cells were centrifuged at 18,000 g for 20 min at 4°C, and the supernatant was collected. The total protein concentration of cell lysed supernatant was determined using BCA Protein Assay Kit (Thermo Scientific, Rockford, IL, United States) according to the manufacturer’s instruction. For PCNA and CC3 ELISA, the cell lysate was adjusted to 5 μg/ml and 1000 μg/ml, respectively. ELISA assay was performed according to the manufacturer’s instructions.

### *In vitro* wound healing assay

CytoSelect Healing Assay (Cell Biolabs, San Diego, CA, United States) evaluated wound healing *in vitro*. HDFs (0.75 × 10^5^ per/well) were seeded in a 24-well plate with CytoSelect wound healing inserts and cultured overnight in HDF-GM to form a monolayer around the insert. The insert was removed, leaving an approximate 0.9 mm wound field between cells. The cells were treated with HDF-GM, HDF-SFM, hPSC-CM-N 6× or hPSC-CM-H 6×, and CMF 6× and cultured for 1 day in normoxia and hypoxia, respectively. hPSC-CM 6× was diluted from hPSC-CM 10× stock using HDF-SFM. Cytoselect cell stain solution was added to each well for 15 min, washed 3 times with deionized water, and imaged using a light microscope (Olympus, Tokyo, Japan). The percentage of cell migration to the wound field was measured using Image J software (National Institutes of Health, Bethesda, MD) (*n* = 4 per group, 6 random fields per sample).

### *In ovo* chorioallantoic membrane assay

To investigate the angiogenic effect of CMF, CAM Assay was performed *in ovo* (n = 6). Fertilized eggs were obtained from SNL Feeds (Lexington, NC, United States) and developed in the Intelligent Egg Incubator 56S (Ridgeyard, China) at 37°C for 72 h at a constant humidity of 50%. On day 3 of development, 4 ml of albumin was removed from each egg using a sterile 25-gauge needle, and a circular window (1 × 1 cm^2^) was cut into the top of each eggshell. The window was sealed with Tegaderm film (3M Science, Minneapolis, MN, United States), and the eggs were placed into the incubator for an additional 5 days (day 8 of development). Chick embryo and vessel development were observed each day. On day 8 of development (day 0 of CAM assay), CMF 6× (20 μl) and the control (PBS, (20 μl) were mixed and absorbed into a gelatin-based scaffold (5 mm diameter) (Gelfoam^®^, Upjohn, Kalamazoo, MI, United States) respectively, and placed on top of the CAM. The CAM was imaged on day 0 using a stereoscopic zoom microscope (Nikon SMZ-U, Tokyo, JP), and vessels peripheral to the scaffold were counted using Image J software. On day 5 of the CAM assay (day 13 of development), CAM was imaged, and vessels peripheral to the scaffold were counted. The day 5 vessel count was subtracted from the day 0 vessel count to determine the number of newly formed vessels (*n* = 6 per group and time point). Following imaging, day 5 gel foam constructs were harvested, fixed with 4% paraformaldehyde, processed and embedded with paraffin, sectioned at 5 μm, and placed on histology slides. Each sample was sectioned in a horizontal and vertical orientation, stained with hematoxylin and eosin (H&E), and imaged using a light microscope. Stained vessels of the horizontal sections were counted using Image J software (*n* = 4 per group).

### Effects of condition medium factor on endothelial cell growth

The effect of CMF on HUVEC growth was compared with hPSC-derived CM and HUVEC/hPSC co-culture. The HUVEC (5000 cells/well, passage 3) was plated in 24 well plates overnight in HUVEC-GM and then treated with HUVEC serum free-medium [HUVEC-SFM; EBM-2 with 0.1% (w/v) bovine serum albumin (BSA; MP Biomedicals, Solon, OH, United States)], hPSC-CM-N 6× for normoxia, hPSC-CM-H 6× for hypoxia, and CMF 6×, respectively. For the co-culture group, hPSC (0.315 x 106 cells) were plated in transwell inserts (Transwell Permeable Supports; Corning, NY, United States) in hPSC-GM. For the hPSC-CM groups, HUVEC-SFM was used to dilute hPSC-CM 10× stock to hPSC-CM 6×. The hPSC-seeding density used for the co-culture and CM collection was the same. Following overnight culture, the hPSC-seeded insert was combined with the HUVEC-seeded 24 well plates and cultured in a HUVEC-SFM medium. The test samples were cultured in normoxia (21% O_2_) and hypoxia (1% O2). AlamarBlue assay was performed on days 0, 2, and 3. The OD value of each sample was converted to the cell number using a standard curve. The cell number of each group and time point was normalized to the cell number at day 0. The standard curve was developed with the seeding density of 0, 3125, 6250, 12,500, 12,500, 25,00, 50,000, 100,000, 200,000 cells/well in 24 well plates (n = 4 per group and time point, duplicate measure).

### Statistical analysis

Results were analyzed with Origin Pro 8.5 (OriginLab Co, Northampton, MA), GraphPad Prism 8 (GraphPad Software, San Diego, CA, United States), and SPSS software (SPSS, version 19; IBM, Armonk, NY, Unites States). T-test, one-way or two-way analysis of variance (ANOVA), and Tukey’s post hoc tests were applied to mean comparisons. Variables are expressed as a mean ± standard deviation (SD), and differences between experimental groups were considered statistically significant at *p*  <  0.05.

## Results

### hPSC-derived conditioned medium analysis

The secretome profile was obtained by proteomic analysis of hPSC-CM obtained under normoxic and hypoxic conditions (hPSC-CM collected from 3 different donors, *n* = 3 samples from each batch of hPSC-CM) (Supplementary Material). Nine proteins that promote wound healing and angiogenesis and are expressed at higher than normal serum levels in hPSC-CM-N and hPSC-CM-H (Sakamoto et al., 1996; Kyrtsonis et al., 1998; Miyake et al., 1999; Di Raimondo et al., 2001; Malatino et al., 2003; Nockowski et al., 2004; Anargyrou et al., 2008; Albanes et al., 2009; Jing et al., 2012; Ren et al., 2012; Kleiner et al., 2013; Moon et al., 2014; Noman et al., 2017; Kritmetapak et al., 2021; Said et al., 2021) were selected. Any proteins possessing inhibitory effects for wound healing were excluded from consideration in this study. The concentration and major roles of these nine proteins are listed in Figure 1B. ANG-1, FGF-7, Follistatin, HGF, IL-6, TGFβ-1, and uPAR, were highly expressed in both normoxic and hypoxic hPSC-CM; the protein concentration in normoxic hPSC-CM and hypoxic hPSC-CM was not statistically different (*p* < 0.05, Student *t*-test). Insulin was highly expressed in both normoxic and hypoxic hPSC-CM, but the concentration of Insulin was significantly higher in hypoxic hPSC-CM than in normoxic hPSC-CM (*p* < 0.05, Student *t*-test). In addition, VEGF was highly expressed in hypoxic hPSC-CM but was not detected in normoxic hPSC-CM. These nine proteins are associated with accelerating wound healing and/or angiogenesis. The major role of FGF-7 (Werner and Grose, 2003; Barrientos et al., 2008; Grada and Falanga, 2018; Huang et al., 2020), Follistatin (McDowall et al., 2008; de Kretser et al., 2012; Ham et al., 2021), IL-6 (Werner and Grose, 2003; Ferreira and Gomes, 2018; Johnson et al., 2020), TGFβ-1 (Clark et al., 1997; Barrientos et al., 2008; Pakyari et al., 2013; Liu et al., 2016; Ahangar et al., 2020), and uPAR (Solberg et al., 2001; Blasi and Carmeliet, 2002; Li et al., 2003; D'Alessio et al., 2008; Rao et al., 2013; Cooper et al., 2018; Mahmood et al., 2018) includes promoting re-epithelialization, extracellular matrix production and wound closure. ANG-1 (Werner and Grose, 2003; Ferreira and Gomes, 2018; Grada and Falanga, 2018; Ahangar et al., 2020), HGF (Werner and Grose, 2003; Ferreira and Gomes, 2018; Grada and Falanga, 2018; Park et al., 2018; Huang et al., 2020), Insulin (Liu et al., 2009; Escudero et al., 2017), and VEGF (Werner and Grose, 2003; Ferreira and Gomes, 2018; Grada and Falanga, 2018; Ahangar et al., 2020; Huang et al., 2020) promote angiogenesis. Based on the proteomics study, the composition and concentration of nine recombinant proteins as a wound healing therapy (CMF) were determined. The normoxic hPSC-CM concentrations in Figure 1B for all recombinant proteins except Insulin and VEGF were selected for use in this study. As Insulin and VEGF concentrations were higher in hypoxic hPSC-CM, the recombinant Insulin and VEGF concentrations selected were the same concentration as in hypoxic hPSC-CM (Figure 1B).

### Optimization of condition medium factor concentration

We investigated the effect of CMF concentration on HDF growth to determine the optimal CMF concentration. HDF was cultured in various concentrations of CMF in normoxia and hypoxia, respectively **(** Figure 2A **)**, and cell number was calculated on days 0, 2, and 5 (*n* = 3, two-way ANOVA and Tukey’s test) **(** Figures 2B,C **)**. No significant difference was detected in the HDF number at day 0 in normoxia and hypoxia (*p* > 0.05). In normoxia, all the CMF treatment groups (CMF 1×, 2×, 4× 6×, 8×, and 10×) showed significantly increased cell numbers compared with SFM (CMF 0×) on days 2 and 5 (*p < 0.05) (Figure 2B). HDF number of CMF 8× was significantly increased compared with CMF 1× (^†^ *p* < 0.05) on day 5. HDF number of CMF 10× was higher than CMF 1× (^†^ *p* < 0.05), 2×, 4×, and 6× on day 2 and day 5 (^‡^ *p* < 0.05). In hypoxia, CMF 4× 6×, 8×, and 10× showed significantly increased HDF number compared with SFM (*p < 0.05), CMF 1×, and 2× (^†^ *p* < 0.05) on day 2; however, there was no significant difference between CMF 4× 6×, 8×, and 10× (*p* > 0.05) (Figure 2C). On day 5 in hypoxia, significantly increased cell number was detected in CMF 6× and 8× treatments in comparison to SFM (*p < 0.05) and CMF 1× (^†^ *p* < 0.05) (Figure 2C). CMF 8× showed a higher cell number than CMF 2× (^‡^ *p* < 0.05). However, no significant difference was found between CMF 6× and 8× (*p* > 0.05). Unexpectedly, the HDF number of CMF 10× decreased compared with the other groups on day 5 in hypoxia, indicating possible inhibitory effects of CMF 10× treatment in hypoxia.

To investigate further the relationship between CMF concentration and cell growth, linear regression analysis was performed. The CMF 10× concentration in hypoxia was excluded in this analysis due to the inhibitory effect on cell growth observed above. Linear regression indicates a strong positive relationship between CMF concentration and cell number, showing R^2^ > 0.7031 both in normoxia and hypoxia (Figures 2D,E). These results indicate that CMF increases cell growth in a dose-dependent manner but not in concentrations greater than 6× (*p* > 0.05 between CMF 6× and CMF 8×) (Figure 2C). Overall, CMF at a concentration of 6× is the optimal concentration to enhance HDF growth and was selected and denoted as CMF throughout the remaining studies.

### Effects of recombinant protein combination (condition medium factor) compared with individual proteins

**FIGURE 3:**
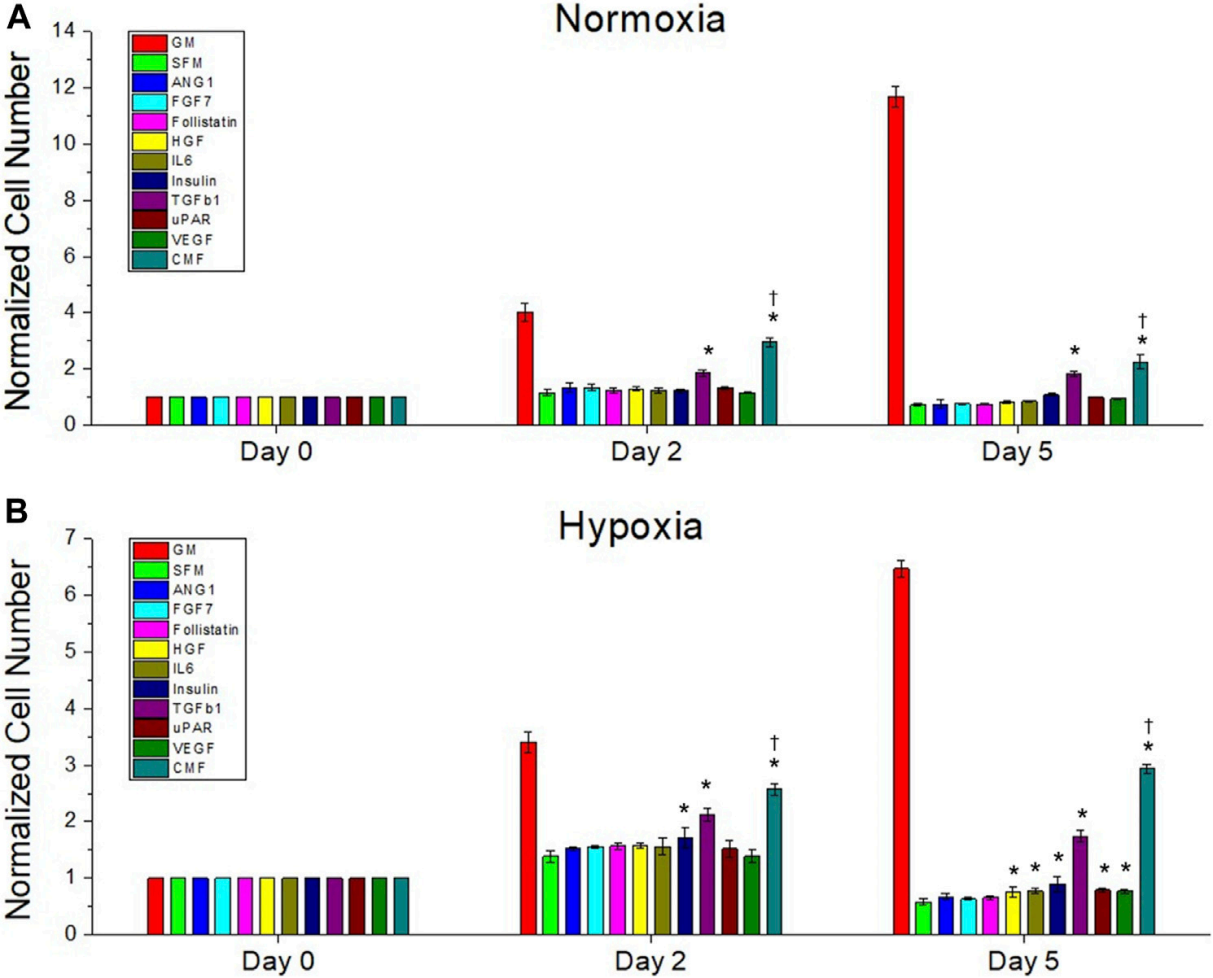
The effect of recombinant protein combination (CMF) on HDF growth compared with individual protein. Treatment with individual proteins (ANG-1, FGF-7, Follistatin, HGF, IL-6, Insulin, TGFβ-1, uPAR, or VEGF) or their combination (CMF - ANG-1, FGF-7, Follistatin, HGF, IL-6, Insulin, TGFβ-1, uPAR, and VEGF) for 0, 2, and 5 days culture under normoxic **(A)** and hypoxic **(B)** conditions. The cell number of each group is measured using AlamarBlue assay and normalized to the day 0 cell number. The CMF shows significantly increased cell number compared with all individual proteins (^†^ *p* < 0.05) at day 2 and day 5 in normoxia and hypoxia (*n* = 3, duplicate measure, two-way ANOVA and Tukey’s test, *p < 0.05 with SFM, ^†^ *p* < 0.05 with ANG-1, FGF-7, Follistatin, HGF, IL-6, Insulin, TGFβ-1, uPAR, and VEGF).

We compared the effect of recombinant protein combination CMF (CMF 6×) (ANG-1, FGF-7, Follistatin, HGF, IL-6, Insulin, TGFβ-1, uPAR, and VEGF) and individual proteins on HDF growth (*n* = 3, two-way ANOVA and Tukey’s test) (Figure 3). On day 0 in normoxia and hypoxia, no significant difference was detected in HDF proliferation (*p* > 0.05). On day 2 and day 5, in normoxia and hypoxia, TGFβ-1 and CMF showed significantly increased cell numbers compared with SFM (*p < 0.05). The CMFgroup showed significantly increased cell numbers compared with all individual proteins (^†^ *p* < 0.05) on days 2 and 5 in hypoxia and normoxia conditions. On day 5, the HDF number of the CMF group increased 3-fold in normoxia and 5-fold in hypoxia compared with SFM. These results indicate that treatment with CMF yields higher cell growth than individual proteins.

### Effects of condition medium factor compared with conditioned medium and hPSC co-culture

**FIGURE 4:**
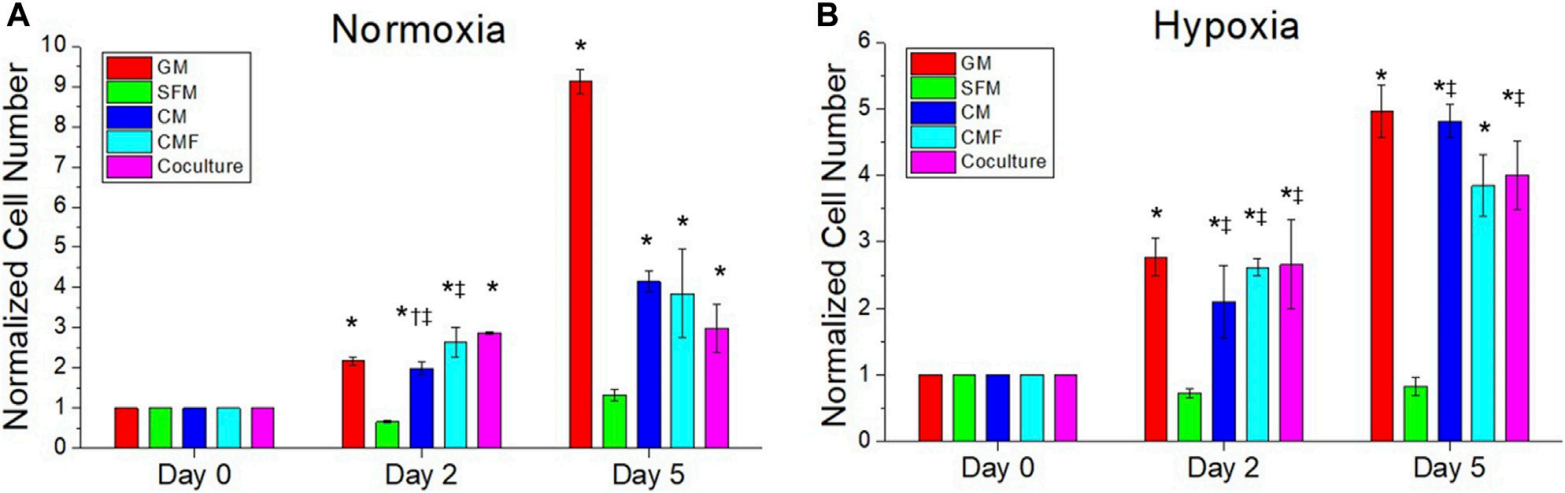
The efficacy of CMF compared with CM and hPSC co-culture. HDF is treated with growth medium (GM), SFM, CM (6×), CMF (6×), and hPSC (co-culture with hPSC) for 0, 2, and 5 days in normoxia **(A)** and hypoxia **(B)**. Normalized cell numbers are measured using AlamarBlue assay. CMF has a similar effect to CM and co-culture treatment on HDF growth (*n* = 3, duplicate measure, two-way ANOVA and Tukey’s test, *p < 0.05 with SFM, ^†^ *p* < 0.05 with CMF, ^‡^ *p* > 0.05 with GM).

The effect of CMF, CM, and hPSC/HDF co-culture on HDF growth was investigated. HDF was cultured in normoxia and hypoxia with HDF-GM, SFM, hPSC-CM 6×, CMF 6×, and hPSC-HDF co-culture (*n* = 3, two-way ANOVA and Tukey’s test) (Figure 4). HDF growth rates increased significantly with the treatment of CM, CMF, and co-culture compared to SFM on days 2 and 5 in normoxia and hypoxia (*p < 0.05). CMF increased HDF number 3-fold and 5-fold compared to SFM at day 5 in normoxia and hypoxia (Figures 4A,B), respectively. No significant difference was detected between CM, CMF, and co-culture treatment groups on day 2 in hypoxia and day 5 in normoxia and hypoxia (*p* > 0.05). These results indicate that CMF has a similar effect to CM and co-culture treatment on HDF growth.

### Human dermal fibroblasts proliferation and apoptosis by condition medium factor treatment

**FIGURE 5:**
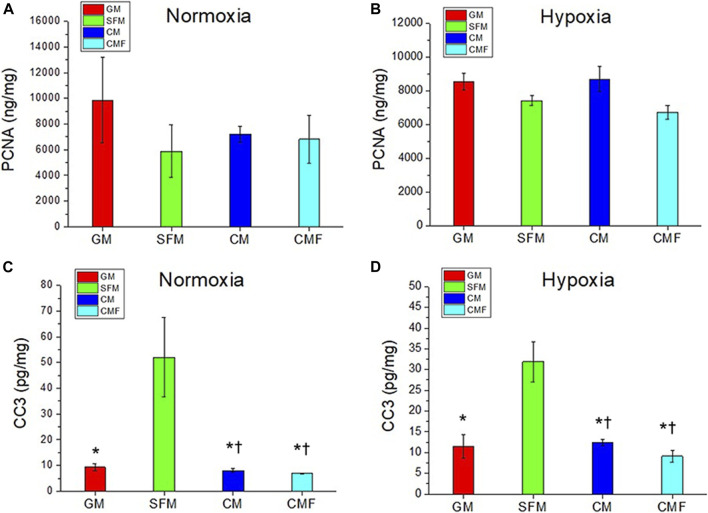
HDF proliferation and anti-apoptosis effect of CMF treatment. HDF is treated with GM, SFM, CM (6×), and CMF (6×) for 3 days, and pro-proliferative and anti-apoptotic effect is investigated using Proliferating cell nuclear antigen (PCNA) and Cleaved caspase-3 (CC3) ELISA assay. **(A,B)** PCNA (ng/mg) in normoxia **(A)** and hypoxia **(B)**. **(C,D)** CC3 (pg/mg) in normoxia **(C)** and hypoxia **(D)**. PCNA protein levels show no significant difference between GM, SFM, CM, and CMF. CMF and CM prevent HDF apoptosis but do not improve proliferation significantly (*n* = 3, one-way ANOVA and Tukey’s test, * *p* < 0.05 with SFM, ^†^ *p* > 0.05 with GM).

The pro-proliferative and anti-apoptotic effects of CMF were investigated using HDF. PCNA ELISA assay was performed to evaluate proliferation (*n* = 3, one-way ANOVA and Tukey’s test) (Figures 5A,B). No significant difference was detected in PCNA protein levels between SFM, CM, and CMF groups in normoxia and hypoxia (*p* > 0.05). CC3 ELISA assay was performed to evaluate apoptosis (*n* = 3, one-way ANOVA and Tukey’s test) (Figures 5C,D). CC3 protein levels in HDF treated with CM and CMF were significantly decreased compared with SFM in normoxia and hypoxia (*p < 0.05) and not statistically different compared with GM (^†^ *p* > 0.05). CC3 level with CMF treatment was decreased 3-fold in normoxia and 7-fold in hypoxia. CM and CMF showed no statistical difference in HDF apoptosis (*p* > 0.05). These results indicate that CMF and CM prevent apoptosis but do not improve proliferation significantly.

### Human dermal fibroblasts migration and wound closure by condition medium factor treatment *in vitro*

**FIGURE 6:**
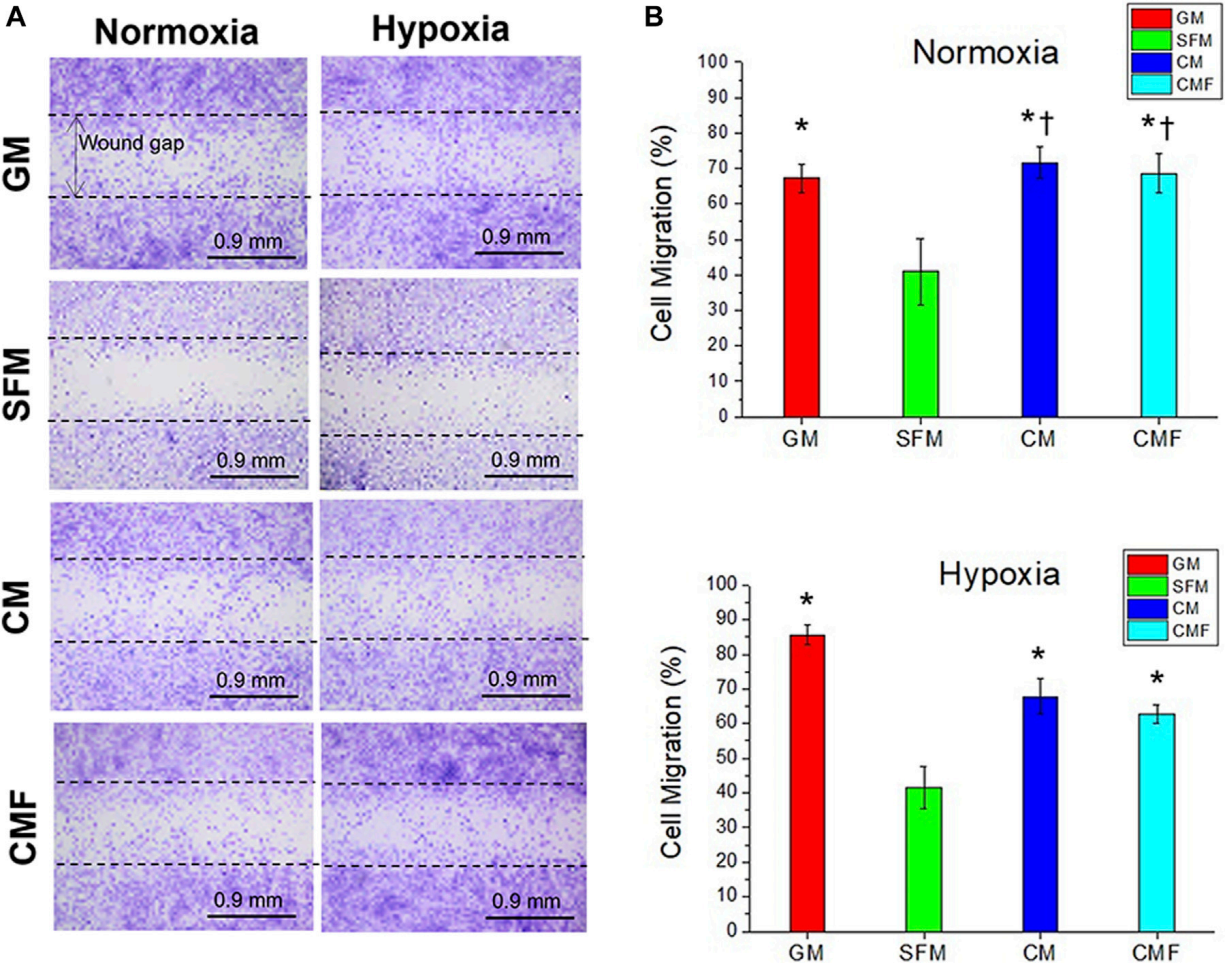
*In Vitro* wound healing assay. HDF migration to the wound gap after 1 day of treatment with GM, SFM, CM (6×), and CMF (6×) in normoxia and hypoxia. **(A)** Representative images of Cytoselect cell staining. **(B)** Cell Migration (%). CMF accelerates cell migration and wound closure (*n* = 4 per group, 6 random fields per sample, one-way ANOVA and Tukey’s test, *p < 0.05 with SFM. ^†^ *p* > 0.05 with GM). Dash lines indicate the initial wound gap (0.9 mm). Scale bars: 0.9 mm.

The effect of CMF on cell migration and wound closure was evaluated *in vitro* using CytoSelect Wound Healing Assay (*n* = 4 per group, 6 random fields per sample, one-way ANOVA, and Tukey’s test) (Figures 6A,B). Cell migration (%) of GM, CM, and CMF increased significantly, approximately 2-fold, compared with SFM in normoxia and hypoxia (*p < 0.05). There was no statistically significant wound closure effect between CM and CMF (*p* > 0.05). These results indicate that CMF and CM positively affect HDF migration and wound closure with no statistical difference.

### Promoting angiogenesis by condition medium factor treatment

**FIGURE 7:**
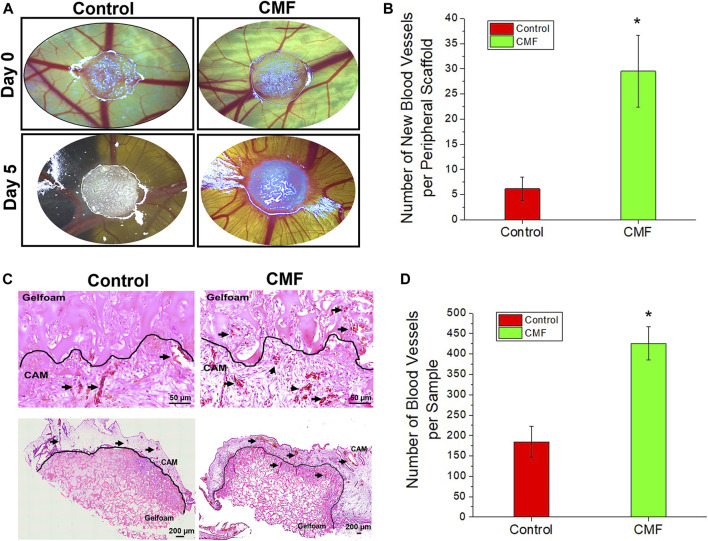
Angiogenic properties of CMF in in ovo chick chorioallantoic membrane (CAM) assay. Gelfoam scaffold treated with CMF (6×) and Control (PBS) is implanted on CAM,incubated in ovo for 5 days, and blood vessel formation is evaluated. **(A)** Brightfield images of CAM model at day 0 and day 5. **(B)** Quantitative analysis of newly formed blood vessel peripheral to scaffold after 5 days implantation (*n* = 6 per group, T-Test, and Welche’s correction *p < 0.05 with control). **(C)** Histological analysis of vertical and horizontal sections through CAM. Upper panel: H&E (Scale bars: 50 μm) of vertical sections. Bottom panel: H&E (Scale bars: 200 μm) of horizontal sections. **(D)** Quantitative analysis of blood vessel per H&E day 5 images (*n* = 4 per group, T-Test, and Welch’s correction *p < 0.05 with control).

The angiogenic effect of CMF was investigated *in ovo* using chick CAM assay (*n* = 6 per group, T-test, and Welch’s correction). Gelfoam containing CMF and the PBS (control) on CAM was imaged on days 0 and 5 after implantation. (Figure 7A) the newly formed blood vessel number of the CMF group increased significantly, approximately 5-fold, compared with the control group (*p < 0.05) (Figure 7B). These results indicate that treatment with CMF yields a higher blood vessel number than the control group. On day 5, gelfoam constructs with surrounding CAM were harvested and processed histologically. H&E staining of the vertical and horizontal sections shows vessel formation in the CAM and gelfoam area of the CMF group but not in the gelfoam area of the control treatment group (Figure 7C—arrows). Blood vessel number per H&E sample of the CMF group increased significantly, approximately 2-fold, compared with the control (Figure 7D) (*n* = 4 per group, T-test, and Welch’s correction, * *p* < 0.05). These results indicate that treatment with CMF stimulates neovascularization and blood vessel formation in the scaffold compared to the control group.

### Human umbilical vein endothelial cell growth by condition medium factor treatment

**FIGURE 8:**
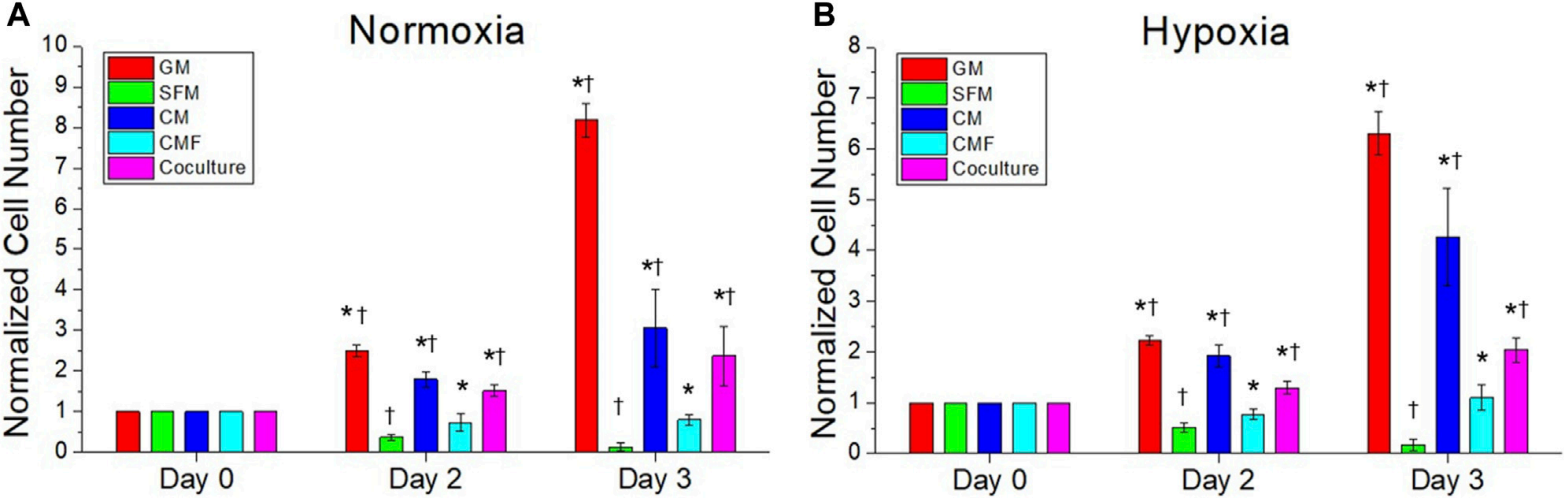
The efficacy of CMF compared with CM and hPSC co-culture on human umbilical vein endothelial cell (HUVEC) growth. HUVECs are cultured in normoxia **(A)** and hypoxia **(B)** with HUVEC-GM, HUVEC-SFM, hPSC-CM 6×, CMF 6×, and hPSC-HUVEC co-culture. Cell number is calculated using AlamarBlue assay and normalized to the cell number at day 0. HUVEC number treated with CMF is significantly higher than SFM but lower than CM and co-culture (*n* = 4, duplicate measure, two-way ANOVA and Tukey’s test, *p < 0.05 with SFM, ^†^ *p* < 0.05 with CMF).

The effect of CMF, CM, and hPSC/HUVEC co-culture on HUVEC growth was investigated. HUVEC was cultured in normoxia and hypoxia with HUVEC-GM, HUVEC-SFM, hPSC-CM (6×), CMF (6×), and hPSC-HUVEC co-culture (*n* = 3, two-way ANOVA and Tukey’s test) (Figure 8). No significant difference was detected in the HUVEC number at day 0 in normoxia and hypoxia (*p* > 0.05). HUVEC growth increased significantly with the treatment of CMF, CM, and co-cultured compared to SFM on days 2 and 3 in normoxia and hypoxia (*p < 0.05) (Figure 8). CMF increased HUVEC number 6-fold compared to SFM on day 3 in normoxia and hypoxia. CM increased HUVEC number 42-fold and 24-fold compared to SFM at day 3 in normoxia and hypoxia, respectively. Co-culture increased HUVEC number 17-fold and 11-fold compared to SFM at day 3 in normoxia and hypoxia, respectively. These results indicate that CMF significantly increases HUVEC growth but is less effective than the CM and co-culture groups.

## Discussion

Stem cell-derived secretomes (CM) have been proposed as an alternate treatment modality to stem cell therapy for wound healing applications. However, stem cell-derived secretomes as a treatment modality is challenged by the safety concerns due to the undefined biomolecules in CM. To that end, we developed an effective strategy to identify the prominent factors in CM that contribute to tissue regeneration and wound healing. In this study, nine defined recombinant proteins were selected to develop a therapeutic cocktail (CMF) that promotes cell growth, accelerates cell migration and wound closure, prevents apoptosis in human dermal cells, and enhances angiogenesis associated with wound healing.

In this study, we performed a proteomic analysis of hPSC-derived CM to investigate major secretomes associated with wound healing and skin regeneration. We analyzed 200 human proteins in hPSC-derived CM cultured under normoxia and hypoxia (Supplementary Material). The proteomics results showed that the effective concentrations of ANG-1, FGF-7, Follistatin, HGF, IL-6, TGFβ-1, and uPAR were produced from hPSCs in both normoxic CM and hypoxic CM. These proteins play an important role in enhancing re-epithelization, angiogenesis, and wound closure (Clark et al., 1997; Solberg et al., 2001; Blasi and Carmeliet, 2002; Li et al., 2003; Werner and Grose, 2003; Barrientos et al., 2008; D'Alessio et al., 2008; McDowall et al., 2008; de Kretser et al., 2012; Pakyari et al., 2013; Rao et al., 2013; Liu et al., 2016; Grada and Falanga, 2018; Cooper et al., 2018; Ferreira and Gomes, 2018; Mahmood et al., 2018; Park et al., 2018; Ahangar et al., 2020; Huang et al., 2020; Johnson et al., 2020; Ham et al., 2021). Interestingly, Insulin and VEGF concentrations were higher in hypoxic CM than in normoxic CM. It is known that Insulin and VEGF expressions are up-regulated in hypoxia and stimulate angiogenesis by promoting endothelial cell survival, proliferation, migration, and remodeling (Werner and Grose, 2003; Das et al., 2010; Escudero et al., 2017; Ferreira and Gomes, 2018; Grada and Falanga, 2018; Ahangar et al., 2020; Huang et al., 2020). Previous studies also demonstrated that hypoxic CM enhanced wound closure compared with normoxic CM and found that hypoxic CM contains higher amounts of proteins, e.g., bFGF, VEGF, IL-6, and TGFβ-1 (Chen et al., 2014). Based on these results with a given concentration of each protein, we developed a specific combination of recombinant proteins (CMF; ANG-1, FGF-7, Follistatin, HGF, IL-6, Insulin, TGFβ-1, uPAR, and VEGF) for wound healing therapy.

Several studies showed significantly different secretome profiles depending on cell sources (Kehl et al., 2019; Matz et al., 2020; Shin et al., 2021). For instance, our previous study investigated the secreted proteins in the CM of human cells derived from four different sources, hPSC, AFSC, adipose-derived stem cells (ADSC), and HUVEC, showing different protein secretion depending on cell sources (Matz et al., 2020). Interestingly, a large number of angiogenic and anti-apoptotic proteins with high concentrations were secreted from hPSC compared to the others (e.g., IL-6, HGF, and FGF-7). Our previous study showed that hPSC secreted follistatin, uPAR, and HGF with high concentration (Kim et al., 2022). Another study by others also showed that the major proteins in hPSC-CM included HGF, TGFβ, and IL-6 (Shin et al., 2021). The present study also obtained similar results as the proteins mentioned above were highly expressed in the hPSC-CM in this study.

The optimal CMF concentration was determined in terms of HDF growth. Our CMF concentration study showed that CMF increased HDF growth in a dose-dependent manner but not in high concentrations (>6×). We observed that the CMF treatment with high concentrations, especially CMF 10×, inhibited the HDF growth in hypoxia. It is plausible that this inhibitory effect is due to the multifunctional role of some proteins. For example, IL-6 and TGFβ-1 not only enhance cell survival and proliferation but also induce apoptosis depending on the environment (Moodley et al., 2003; Sanchez-Capelo, 2005). As CMF at a concentration of 6× gave the best cell growth result, CMF 6× was used for further studies. We then compared the HDF growth between nine proteins combination (CMF) and individual proteins. The data showed that the CMF was more effective on HDF growth than the individual proteins.

This study demonstrates that the CMF is as effective as the stem cell-based therapies for accelerating wound healing. CMF at the optimized concentration (6×) supported HDF growth and had similar effects as CM (6×) and hPSC co-culture. In addition, the CMF showed similar anti-apoptosis and wound closure effects to the CM. Interestingly, the CM and CMF did not promote HDF proliferation but did reduce apoptosis.

We also demonstrated the angiogenic effects of the CMF. As discussed above, wound sites are often hypoxic due to the loss of blood supply caused by trauma, burn, or injury. Furthermore, the proliferation phase of wound healing is highly metabolic with increased demand for oxygen and nutrients, requiring re-epithelialization, granulation tissue formation, and scarless healing (Landen et al., 2016). Therefore, restoring blood supply to the wound sites through promoting angiogenesis is critically important in improving wound outcomes. The *in ovo* CAM assay results showed that the CMF enhanced new vessel formation from pre-existing vessels. We also investigated the effect of CMF on HUVECs. The CMF treatment increased HUVEC growth compared with SFM; however, the CMF was not as effective as the CM. It is speculated that several proteins present in the CM but not included in the CMF composition also affected HUVEC growth, e.g., angiogenin, SDF-1, IL-8 (Gillitzer and Goebeler, 2001; Tello-Montoliu et al., 2006; Deshane et al., 2007). Other proteins not included in this study will need to be tested in subsequent studies to improve the angiogenic effects. In addition, the angiogenic effects of the CMF need to be further evaluated using various angiogenesis assays.

Our strategy of using recombinant proteins (CMF), having similar therapeutic effects to cell-based therapy, provides many advantages over cell-based therapies. This treatment modality uses commercially available recombinant proteins with defined composition and concentration. Thus, consistent product quality can be achieved, and safety and regulatory concerns can be minimized and further verified. This strategy can save time, effort, and resources by removing massive cell culture and CM collection processes. More importantly, our treatment modality as an off-the-shelf product facilitates immediate care when desired for traumatic wounds and burns. In addition, the major role of these proteins is promoting growth/proliferation, preventing apoptosis of several cell types not limited to skin cells, and promoting angiogenesis, which is critical for several types of tissue regeneration. Therefore, we expect this treatment modality to be applied to several tissue types for regeneration and reconstruction. For instance, we previously developed a specific combination of recombinant proteins by hPSC-CM analysis to treat kidney diseases (Kim et al., 2022). We identified five major proteins (Follistatin, uPAR, ANGPTL4, HGF, and VEGF) that promote kidney repair in the hPSC-CM. The intrarenal delivery of these proteins ameliorated renal damage and accelerated renal function recovery in a rat acute kidney injury model. The treatment effects were similar to the CM treatment. These results indicate the potential of using recombinant proteins identified by CM analysis as an alternative to the CM to treat damaged or injured tissues.

Although this study showed the feasibility of using the CMF to accelerate wound healing, several issues need to be addressed to improve the therapeutic outcomes. This study focused on evaluating the effects of CMF using HDF, a representative cell type of the dermis, as the regeneration of the dermis is challenging compared with the epidermis layers (Zhou et al., 2017). The treatment effects need to be validated and optimized with not only endothelial cells but also other cell types, e.g., keratinocytes and immune cells. Subsequent studies will investigate protein combinations to improve treatment efficacy, including other proteins not tested in this study as discussed above, while reducing the total number of proteins in our recombinant cocktail. For efficient delivery of recombinant proteins *in vivo*, developing a delivery system capable of controlled delivery and sustained release is necessary. *In vivo* feasibility and efficacy of the CMF combined with the delivery system will be investigated in future studies in an animal model of full-thickness skin wounds.

## Conclusion

This study demonstrated the feasibility of using recombinant proteins (CMF) identified by stem cell-CM analysis for wound healing applications. The CMF enhances HDF survival and growth and accelerates wound closure *in vitro*. The CMF treatment was as effective as stem cell and CM treatments, showing a future potential alternative with further advances. In addition, the CMF promotes angiogenesis. Future work will investigate the wound healing effect of CMF *in vivo*. Our strategy may provide an efficient therapeutic solution by offering off-the-shelf products for immediate wound care and healing.

## Data Availability

The raw data supporting the conclusions of this article will be made available by the authors, without undue reservation.
