# Supplementary material for: Identification and characterization of stem cell secretome-based recombinant proteins for wound healing applications
Source: Front Bioeng Biotechnol. 2022 Jul 22;10:954682. doi: 10.3389/fbioe.2022.954682 (PMC9354600; doi:10.3389/fbioe.2022.954682)
Supplement: Supplementary file 1 [file DataSheet1.PDF]

Supplementary Material

Supplementary material. Identification of human proteins by proteomics of human placental stem cell-derived conditioned medium (CM 4×) in normoxia and hypoxia. *LOD (value below the limit of detection). **MAX (value above the highest standards)

CM 4x (pg/ml)	Normoxia			Hypoxia		
	Donor 1	Donor 2	Donor 3	Donor 1	Donor 2	Donor 3
**6Ckine**	10305.0	9438.8	4960.8	9684.7	7095.4	5096.2
**Axl**	855.7	1327.3	827.3	466.5	935.3	621.4
**BTC**	67.0	328.8	55.2	83.0	61.2	0.0*
**CCL28**	0.0*	973.4	518.5	0.0*	482.8	0.0*
**CTACK**	2646.0	3052.1	1839.5	888.1	2670.4	85.5*
**CXCL16**	837.6	937.6	329.6	506.5	390.8	210.1
**ENA-78**	2407.6	4997.5	459.7	1700.0	2835.2	337.0
**Eotaxin-3**	522.3	46.5	242.9	0.0*	152.1	0.0*
**GCP-2**	1381.9**	1254.9**	429.5	1430.8**	1075.1**	500.8
**GRO**	292.7	133.9	6.9	49.1	51.1	0.0*
**HCC-1**	0.0*	0.0*	0.0*	0.0*	0.0*	0.0*
**HCC-4**	6.0*	17.6	0.0*	8.0	0.2*	0.0*
**IL-9**	0.0*	2322.8	0.0*	8655.7	94.3*	0.0*
**IL-17F**	0.0*	13.2*	0.0*	10.0*	0.0*	0.0*
**IL-18 BPa**	901.1*	2003.4	0.0*	2554.4	2993.9	0.0*
**IL-28A**	105.0	155.2	102.4	353.1	279.3	51.8
**IL-29**	532.1*	5500.7	0.0*	115.4*	0.0*	2033.8
**IL-31**	32.2	4.1*	0.0*	26.8	7.9*	0.0*
**IP-10**	0.0*	72.8	0.0*	18.1	6.1	0.0*
**I-TAC**	1109.3	1463.2	1077.2	708.9	1064.6	332.4
**LIF**	912.4	1037.1	470.5	539.4	1515.3	215.8
**LIGHT**	254.4	521.0	143.2	347.9	225.0	222.7
**Lymphotactin**	4709.7	4733.9	3918.3	4409.6	5789.8	4680.6
**MCP-2**	573.2	174.0	10.5	144.7	40.5	4.9
**MCP-3**	1750.7	1646.7	89.4	1030.5	783.5	90.5

**MCP-4**	56.6	9.2	0.0*	19.9	6.2	0.0*
**MDC**	9.2	0.0*	0.0*	0.0*	3.4	0.0*
**MIF**	5478.8	7257.7	4089.5	3253.3	2368.3	1213.9
**MIP-3a**	26.1	64.3	2.2	107.6	217.6	5.6
**MIP-3b**	24.4*	20.7*	0.0*	25.7*	53.9	0.0*
**MPIF-1**	0.0*	15.1*	0.0*	402.5	37.0*	0.0*
**MSP**	98.1*	349.3	0.0*	13.0*	84.1*	0.0*
**NAP-2**	5.4	10.8	1.9	10.5	3.5	1.1
**OPN**	12049.3	31754.8	65393.6	7626.3	27519.5	52091.0
**PARC**	0.1*	5.3	0.0*	0.0*	0.0*	0.0*
**PF4**	10342.7	5525.5	741.9	9594.9	5100.4	696.8
**SDF-1a**	217.1	214.2	218.2	63.0	249.0	169.9
**TARC**	1.4*	12.1	0.0*	7.2	21.3	0.0*
**TECK**	0.0*	545.2	0.0*	0.0*	1015.6	0.0*
**TSLP**	0.0*	0.0*	0.0*	0.0*	3.1*	0.0*
**Activin A**	4593.8	152.6	0.0*	263.4	611.8	101.5
**AgRP**	22.4*	88.6	0.0*	66.9	0.0*	0.0*
**Angiogenin**	1451.0**	1370.4**	1040.9	1685.6**	1905.7**	1660.1**
**ANG-1**	6474.6	3113.2	3081.1	2674.7	2015.3	2411.4
**Angiostatin**	9008.1	0.0*	0.0*	3724.2*	0.0*	0.0*
**Cathepsin S**	2035.3	576.8	39.0	242.3	18.8	0.0*
**CD40**	0.0*	0.0*	0.0*	0.0*	0.0*	0.0*
**Cripto-1**	0.0*	0.0*	0.0*	0.0*	0.0*	0.0*
**DAN**	0.0*	0.0*	0.0*	0.0*	0.0*	0.0*
**DKK-1**	7404.5	19523.1	14446.6	4414.6	13597.9	11439.0
**E-Cadherin**	0.0*	0.0*	0.0*	0.0*	0.0*	0.0*
**EpCAM**	25.1	0.0*	0.0*	0.0*	0.0*	0.0*
**FAS L**	0.0*	0.0*	0.0*	341.6	0.0*	0.0*
**Fcg RIIBC**	0.0*	0.0*	0.0*	0.0*	0.0*	0.0*
**Follistatin**	6415.8	11904.1	14835.3	9463.3	7090.4	11934.2
**Galectin-7**	0.0*	0.0*	0.0*	0.0*	0.0*	0.0*
**ICAM-2**	0.0*	0.0*	0.0*	0.0*	0.0*	0.0*
**IL-13 R1**	0.0*	0.0*	0.0*	0.0*	0.0*	0.0*
**IL-13 R2**	0.0*	0.0*	0.0*	105.7*	0.0*	0.0*
**IL-17B**	265.2	0.0*	0.0*	0.0*	0.0*	184.4*
**IL-2 Ra**	35.9	0.0*	6.6*	100.8	0.0*	2.4*
**IL-2 Rb**	955.5	0.0*	0.0*	1246.8	0.0*	0.0*
**IL-23**	506.7	0.0*	0.0*	623.6	1832.2	0.0*
**LAP(TGFb1)**	1006.2	841.7	764.9	859.9	1388.1	883.5
**NrCAM**	8.4*	0.0*	0.0*	173.9	0.0*	0.0*

**PAI-1**	15498.2	11146.7	10647.2	14162.2	13888.0	11522.7
**PDGF-AB**	0.0*	0.0*	0.0*	0.0*	0.0*	0.0*
**Resistin**	2.2*	0.0*	0.0*	34.7	0.0*	0.0*
**SDF-1b**	138.6	142.1	194.2	0.0*	80.0	177.3
**gp130**	6434.1	7736.8	4374.7	2693.6	3028.3	2619.2
**Shh-N**	137.6	542.5	767.5	188.7	0.0*	381.3
**Siglec-5**	99.0	76.2	0.0*	118.8	0.0*	0.0*
**IL-1 R4**	0.0*	0.0*	0.0*	0.0*	0.0*	0.0*
**TGFb2**	145.7*	0.0*	0.0*	134.2*	0.0*	0.0*
**Tie-2**	45.0*	156.1	0.0*	176.1	0.0*	0.0*
**TPO**	10.3*	0.0*	0.0*	169.6	0.0*	0.0*
**TRAIL R4**	0.0*	0.0*	0.0*	0.0*	0.0*	0.0*
**TREM-1**	59.7*	65.9*	0.0*	0.0*	0.0*	0.0*
**VEGF-C**	33.1	11.7*	53.4	78.6	0.0*	0.0*
**VEGF R1**	43801.2	47264.9	38437.0	14480.0	10485.1	7323.0
**AR**	22.7	1.8*	68.3	0.0*	0.0*	8.4
**BDNF**	590.7**	653.1**	371.4	309.0	642.0**	243.7
**bFGF**	4.5*	56.5	19.0	0.0*	0.0*	0.0*
**BMP-4**	0.0*	0.0*	263.0*	0.0*	0.0*	0.0*
**BMP-5**	7834.1	28196.5	20532.8	0.0*	0.0*	0.0*
**BMP-7**	0.0*	0.0*	204.0*	0.0*	0.0*	0.0*
**b-NGF**	0.0*	0.0*	12.0	0.0*	0.0*	3.6
**EGF**	0.0*	0.0*	0.0*	0.0*	0.0*	0.0*
**EGF R**	372.8	386.5	564.6	205.8	133.6	449.0
**EG-VEGF**	0.0*	0.0*	0.0*	0.0*	0.0*	0.0*
**FGF-4**	0.0*	0.0*	0.0*	0.0*	0.0*	0.0*
**FGF-7**	1518.1	2030.0	763.3	404.4	0.0*	260.7
**GDF-15**	673.5**	549.8**	494.9**	490.0**	180.2**	423.5**
**GDNF**	213.8	94.3	12.3*	45.2	0.0*	0.0*
**GH**	0.0*	0.0*	0.0*	0.0*	0.0*	0.0*
**HB-EGF**	0.0*	0.0*	11.1	14.4	0.0*	0.0*
**HGF**	16334.8**	16334.1**	17341.7**	15205.0**	14162.6**	15316.3**
**IGFBP-1**	0.0*	0.0*	0.0*	0.0*	0.0*	0.0*
**IGFBP-2**	0.0*	473.6	5075.4	0.0*	0.0*	2418.0
**IGFBP-3**	504529.5**	321545.0	146457.8	535792.8**	477613.0**	530127.5**
**IGFBP-4**	351226.2	282398.9	237227.0	351273.3	339920.0	293055.4
**IGFBP-6**	23776.5**	20214.1	26089.2**	24851.4**	19177.3	25905.4**
**IGF-1**	0.0*	0.0*	0.0*	0.0*	0.0*	0.0*
**Insulin**	4789.7	8810.0	5280.5	10318.5	19212.3**	20759.5**
**MCSF R**	0.0*	0.0*	0.0*	160.0	0.0*	0.0*
**NGF R**	0.0*	0.0*	0.0*	0.0*	0.0*	0.0*
**NT-3**	0.0*	0.0*	99.0	0.0*	0.0*	0.0*
**NT-4**	0.0*	0.0*	0.0*	49.7	0.0*	0.0*

**OPG**	766.3	1160.6	1419.4	440.7	554.9	1033.1
**PDGF-AA**	117.3	161.2	380.3	155.2	331.5	482.5
**PIGF**	0.0*	0.0*	0.0*	0.0*	0.0*	0.0*
**SCF**	277.7	139.9	70.8	135.0	0.0*	0.0*
**SCF R**	160.0	290.0	142.9	124.5	83.6	119.1
**TGFa**	0.0*	0.0*	0.0*	0.0*	0.0*	0.0*
**TGFb1**	11836.8	80399.4	32225.9	13882.4	18627.3	12293.9
**TGFb3**	0.0*	0.0*	0.0*	0.0*	0.0*	0.0*
**VEGF**	0.0*	0.0*	0.0*	1508.2	3027.5	2372.9
**VEGF R2**	0.0*	0.0*	0.0*	0.0*	0.0*	0.0*
**VEGF R3**	0.0*	0.0*	0.0*	0.0*	0.0*	0.0*
**VEGF-D**	0.0*	0.0*	0.0*	0.0*	0.0*	0.0*
**BLC**	2.8	3.7	4.3	2.9	4.1	3.6
**Eotaxin**	229.7	177.0	84.8	27.5	7.1	36.1
**Eotaxin-2**	10.0	15.0	15.8	5.5	10.5	14.1
**G-CSF**	199.3	341.8	21.6	410.5	287.9	20.6
**GM-CSF**	70.5	71.3	139.0	60.2	60.3	88.9
**I-309**	9.4	6.0	19.0	4.9	8.9	9.1
**ICAM-1**	1585.1	2011.1	520.8	1156.0	1459.7	366.9
**IFNg**	0.0*	33.9	121.9	0.0*	49.9	17.5
**IL-1a**	72.7	100.5	82.9	100.8	96.1	73.1
**IL-1b**	66.6	45.7	7.2	66.8	24.0	15.4
**IL-1ra**	1139.8	1688.3	1989.4	1427.9	1803.9	2157.7
**IL-2**	213.8	126.7	1297.3	191.3	85.3	1119.6
**IL-4**	28.0	17.2	47.8	41.2	26.8	27.9
**IL-5**	113.5	117.2	276.3	41.6	7.3	275.1
**IL-6**	6278.1**	6269.9**	3568.1	6003.4**	6671.5**	5376.6**
**IL-6R**	40.5	51.7	128.2	14.6	24.7	80.2
**IL-7**	38.9	54.3	67.2	38.1	52.1	57.9
**IL-8**	480.8**	375.7**	35.8	522.0**	449.4**	299.6**
**IL-10**	4.4	9.1	12.3	2.8	8.5	7.4
**IL-11**	517.9	569.2	187.1	277.7	795.9	409.3
**IL-12p40**	8.8	8.7	8.0	6.3	3.1	6.2
**IL-12p70**	0.0	0.0	0.0	0.0	0.0*	0.0
**IL-13**	0.0*	0.0*	11.4	10.8	0.0*	7.5
**IL-15**	11.6	18.7	16.3	2.9	12.9	10.4
**IL-16**	42.1	35.0	49.2	36.0	30.8	37.9
**IL-17**	0.0*	0.0*	0.3*	5.8	0.0*	0.0*
**MCP-1**	1043.3	508.4	1621.0	738.7	1068.9	1219.1
**MCSF**	193.2	289.8	246.6	4.1	3.8	133.9
**MIG**	0.0*	6.4	10.9	5.6	3.4	3.8
**MIP-1a**	31.8	51.3	40.9	18.1	35.1	43.3
**MIP-1b**	5.3	3.2	2.8	5.0	2.5	3.1

**MIP-1d**	0.0*	0.8	0.3	0.3	0.0*	0.3
**PDGF-BB**	15.7	47.1	24.6	16.8	43.4	29.4
**RANTES**	498.0	1347.3	152.4	437.4	1246.3	184.0
**TIMP-1**	3590.5**	3413.6**	3937.5**	4061.5**	3924.4**	4099.8**
**TIMP-2**	10772.2**	10569.4**	8496.6	10351.7**	10188.3**	9391.4**
**TNFa**	72.3	48.7	106.7	57.1	18.2	83.6
**TNFb**	865.0	576.9	1613.5**	608.8	393.8	2257.4**
**TNF RI**	3984.7	4732.2	4173.9	2377.0	2060.0	2185.8
**TNF RII**	3.0	38.7	12.4	8.0	0.3	17.1
**4-1BB**	0.0*	11.6	0.0*	3.8	0.0*	0.0*
**ALCAM**	3714.2	3114.7	4749.4	3015.9	3470.6	3930.5
**B7-1**	0.0*	35.2	0.0*	7.2*	0.0*	0.0*
**BCMA**	6.9	8.9	6.3	0.0*	0.0*	0.0*
**CD14**	816.2**	507.8	95.4	513.3	409.8	96.3
**CD30**	0.0*	0.0*	0.0*	0.0*	0.0*	0.0*
**CD40L**	10.7	36.7	8.4	12.1	0.4*	0.4*
**CEACAM-1**	0.0*	3.5*	0.8*	0.8*	0.0*	6.9*
**DR6**	1542.2	691.4	199.3	591.0	209.9	83.6
**Dtk**	0.0*	75.8	0.0*	13.0	0.0*	0.0*
**Endoglin**	220.7	98.0	83.7	109.0	55.2	59.2
**ErbB3**	0.0*	0.7*	0.0*	0.0*	4.2*	0.0*
**E-Selectin**	0.0*	130.8	0.0*	35.0*	0.0*	0.0*
**Fas**	228.4	332.6	304.9	162.7	295.4	368.1
**Flt-3L**	7.2	13.5	10.5	4.1	5.8	3.9
**GITR**	5.3*	57.4*	0.0*	52.1*	47.3*	0.0*
**HVEM**	0.0*	8.7*	71.1	23.3	11.7*	9.8*
**ICAM-3**	5.5*	89.8	0.0*	23.8	22.7	0.0*
**Contactin-2**	0.0*	0.0*	0.0*	8.2*	0.0*	0.0*
**IL-1 RI**	0.0*	3.7	0.0*	0.0*	0.0*	0.0*
**IL-2 Rg**	0.0*	0.0*	0.0*	1.2	0.0*	0.0*
**IL-10 Rb**	0.0*	5.3*	0.0*	22.5	5.5*	0.0*
**IL-17R**	0.0*	293.2	0.0*	0.0*	0.0*	0.0*
**IL-21R**	0.0*	0.0*	0.0*	0.0*	0.0*	0.0*
**LIMPII**	90.4	106.6	68.9	58.0	124.5	66.4
**Lipocalin-2**	0.0*	0.0*	0.0*	0.0*	0.2*	0.0*
**L-Selectin**	0.0*	0.0*	0.0*	29.1*	12.7*	0.0*
**LYVE-1**	0.0*	0.6*	0.0*	1.3	0.6*	0.0*
**MICA**	61.1	147.2	734.7	26.6	240.6	449.9
**MICB**	40.8*	1131.3	158.3*	385.7	991.8	218.5
**NRG1-b1**	85.3	35.7	1.9*	36.4	10.5	0.0*
**PDGF Rb**	570.4	1131.3	500.5	749.0	1357.1	727.3
**PECAM-1**	0.0*	43.8*	0.0*	0.0*	0.0*	0.0*
**RAGE**	0.4*	25.9	17.6	1.6*	13.9	0.0*

**TIM-1**	0.0*	4.6*	0.0*	0.0*	0.0*	0.0*
**TRAIL R3**	7.6	58.5	30.0	4.0	28.8	13.4
**Trappin-2**	77.6	111.2	0.0*	352.7	82.1	0.0*
**uPAR**	5748.4	5481.5	4438.1	6489.3	9428.0	5985.3
**VCAM-1**	3048.7	5865.5	1674.2	946.9	604.9	522.9
**XEDAR**	0.0*	2.0*	0.0*	0.0*	3.1*	0.0*
